# Supplementary material for: Gender differentials in the timing and prognostic factors of pubarche in Nigeria
Source: PLoS One. 2022 Nov 21;17(11):e0277844. doi: 10.1371/journal.pone.0277844 (PMC9678277; doi:10.1371/journal.pone.0277844)
Supplement: Attachment — Submitted filename: Pubarche Plos 1 rebutal.docx [file pone.0277844.s001.docx]

Respected Editor,

Thank you for your letter and the opportunity to revise manuscript entitled **“**Timing and Prognostic Factors of Pubarche in Nigeria: Parametric Survival Analysis”.

The suggestions offered by the reviewers have been immensely helpful, and we also appreciate your insightful comments on revising the abstract and other aspects of the paper.

I hereby include the reviewer comments below and provided a point-by-point to all the comments of the Editor and the reviewers

The revisions were approved by all the authors. The manuscript has been scrutinized for requirement in the author instructions, as well. We hope the revised manuscript will better suit the Journal.

Thank you

Adeniyi Fagbamigbe

Journal Requirements:

1. When submitting your revision, we need you to address these additional requirements.

Please ensure that your manuscript meets PLOS ONE's style requirements, including those for file naming. The PLOS ONE style templates can be found at 
https://journals.plos.org/plosone/s/file?id=wjVg/PLOSOne_formatting_sample_main_body.pdf and 
<https://journals.plos.org/plosone/s/file?id=ba62/PLOSOne_formatting_sample_title_authors_affiliations.pdf>

**Thank you. We have corrected all the requirements**

2.Thank you for stating in your Funding Statement: 
This research was supported by the Consortium for Advanced Research Training in Africa (CARTA). CARTA is jointly led by the African Population and Health Research Center and the University of the Witwatersrand and funded by the Wellcome Trust (UK) (Grant No: 087547/Z/08/Z), the Carnegie Corporation of New York (Grant No--B 8606.R02), Sida (Grant No:54100029)”. CARTA had trained the first author in research methodologies. The statements made and views expressed are solely the responsibility of the author.)

Please provide an amended statement that declares *all* the funding or sources of support (whether external or internal to your organization) received during this study, as detailed online in our guide for authors at http://journals.plos.org/plosone/s/submit-now.  Please also include the statement “There was no additional external funding received for this study.” in your updated Funding Statement. 
Please include your amended Funding Statement within your cover letter. We will change the online submission form on your behalf.

**Thank you. We have revised the statements L456. It was a support not funding per sai**

3. We note that the grant information you provided in the ‘Funding Information’ and ‘Financial Disclosure’ sections do not match.

When you resubmit, please ensure that you provide the correct grant numbers for the awards you received for your study in the ‘Funding 4 section.

**Thank you. We have revised the statements**

4. We note that the grant information you provided in the ‘Funding Information’ and ‘Financial Disclosure’ sections do not match.

When you resubmit, please ensure that you provide the correct grant numbers for the awards you received for your study in the ‘Funding Information’ section.

**Thank you. We have revised the statements L456- 465**

5. Please review your reference list to ensure that it is complete and correct. If you have cited papers that have been retracted, please include the rationale for doing so in the manuscript text, or remove these references and replace them with relevant current references. Any changes to the reference list should be mentioned in the rebuttal letter that accompanies your revised manuscript. If you need to cite a retracted article, indicate the article’s retracted status in the References list and also include a citation and full reference for the retraction notice.

**Thank you. We have revised the references and updated the links. We used the Mendeley software. All our references were cited and listed at the end.**

**We retracted** (CIA World Factbook, 2017) Ref 29 because same information was in reference 30

Additional Editor Comments:

This paper assessed the timing of pubarche among adolescents and young adults in Nigeria and identified prognostic factors of the timing by obtaining information on youths' sexual and reproductive developments in a population survey among in-school and out-of-school youths aged 15 to 24 years in Nigeria. A total of 1174 boys and 1004 girls provided valid information on pubarche.
Please supplement the following.

Thank you
1. Supplement the theoretical aspects of the discussion.

**We have supplemented the theoretical aspects viz-a-viz our findings. Kindly see L403-L411**
2. Present the overall frame graphic.

**We have provided the overall frame graphic L95-L98**
