# Supplementary material for: Gender differentials in the timing and prognostic factors of pubarche in Nigeria
Source: PLoS One. 2022 Nov 21;17(11):e0277844. doi: 10.1371/journal.pone.0277844 (PMC9678277; doi:10.1371/journal.pone.0277844)
Supplement: Attachment — Submitted filename: Gender differentials in the timing and prognostic factors of pubarche in Nigeria.docx [file pone.0277844.s002.docx]

**Gender differentials in the timing and prognostic factors of pubarche in Nigeria: Generalized gamma survival model**

Thank you for the opportunity to review the manuscript with the above title. The paper is well written. There are a few things that need to be addressed. My concerns are written in green.

Abstract

Paucity of data exists on the timing of puberty, particularly the pubarche, in developing countries, which has hitherto limited the knowledge of the timing of pubarche, and assistance offered by physicians to anxious young people in Nigeria. I think paucity

We hypothesized that the timing of pubarche in Nigeria differs from other countries.

It appears that there is a comparative study between Nigeria and other developing countries. It is better to hypothesize within the geographical divides in Nigeria. Remove other countries. Also, the first sentence should focus on Nigeria.

Our findings will aid medical practitioners in providing appropriate advice and support on pubarche-related issues among adolescents in Nigeria.

One would like to see how the evidence of pubarche anxiousness in relation to request for medical assistance.

Introduction

The need to update knowledge and to inform surrounding adults to ease anxieties that often comes with the unpredictability of age at pubarche necessitated this study.

Is there evidence of the needs and anxieties with regards to pubarche?

The knowledge of risk factors associated with pubarche could help medical practitioners provide evidence-based care and guidance to adolescents and their guardians.

I expected to see evidence of seeking medical assistance in relation to pubarche issues.

We hypothesized that better educational attainment could improve decisions on healthy food that may, in turn, affect the onset of pubarche; pubertal timing has been demonstrated to be a function of nutritional intake during childhood.

The association between place of residence and timing of pubarche can be explained by the differentials in nutritional intake in rural and urban areas. Also, an Indian study reported rural-urban differences in age at pubarche 23. Can the authors elaborate on this study? Was nutrition implicated?

Race and ethnicity have been identified as associated factors with age at pubarche 24. This was ascribed to differences in the levels of metabolism and insulin across different races. This does not seem to be complete.

Thus, we hypothesized that the timing of pubarche in Nigeria differs from other countries and that age of pubarche in the country has changed over time compared with what is available in literature.

Can this hypothesis be based on what is found in Nigeria? Mentioning other countries that are not part of the study will make testing the hypothesis impossible.

**Significance of the study**

Recent reports showed that the lack of information on puberty is detrimental to the reproductive health of the youths. Where is the evidence of this?

Many adolescents transition to adulthood without correct and adequate information on what puberty entails and how it affects their social and cognitive behaviours. This does not seem to belong here.

**Methods: This section is acceptable**

**Study Design**

**The study design was a retrospective nationally representative population survey. What is the name of the survey and the year it was conducted?**

Line 54-56: Although different studies have estimated the timing of pubarche and identified its associated risk factors in most regions of the world 6–10, such studies are scarce for the Nigeria setting. Besides, the available ones are dated 11,12. Do you mean it is outdated?

The Theoretical / Conceptual Framework is appropriate and well-articulated.

The analysis is very informative and well done.

Discussion

We used survival analysis technique to provide updated information on the incidence and timing of puberty among male and female adolescents in Nigeria. This is for the methodology section. It seems that results are being repeated in the discussion.
