# Supplementary material for: Gender differentials in the timing and prognostic factors of pubarche in Nigeria
Source: PLoS One. 2022 Nov 21;17(11):e0277844. doi: 10.1371/journal.pone.0277844 (PMC9678277; doi:10.1371/journal.pone.0277844)
Supplement: Attachment — Submitted filename: Pubarche Plos 3 rebutal.docx [file pone.0277844.s005.docx]

Respected Editor,

Thank you for your letter and the opportunity to revise manuscript entitled **“**Timing and Prognostic Factors of Pubarche in Nigeria: Parametric Survival Analysis”.

The suggestions offered by the reviewers have been immensely helpful, and we also appreciate your insightful comments on revising the abstract and other aspects of the paper.

I hereby include the reviewer comments below and provided a point-by-point to all the comments of the Editor and the reviewers

The revisions were approved by all the authors. The manuscript has been scrutinized for requirement in the author instructions, as well. We hope the revised manuscript will better suit the Journal.

Thank you

Adeniyi Fagbamigbe

Editors Comment:

My main concern is with the data availability. You are saying that the data is available without restrictions but it is not included with the manuscript and there is no link provided to the data location. Also, a google search for the name of the survey did not provide any meaningful link. If you were not involved in data production, it would also help if you provide cite/link to published materials (eg, survey report). As you know authors are required to make all data underlying the findings described fully available, without restriction, and from the time of publication. PLOS allows rare exceptions to address legal and ethical concerns. See the PLOS Data Policy and FAQ for detailed information.

**We have provided the link to the data availability**

**Data is available on request and can be downloaded at https://drive.google.com/file/d/1BSeAN7mG4vOYICD7Wec4UvaSYKUhwoNo/view?usp=share_link**
